# Autologous Unilateral Breast Reconstruction with Venous Supercharged IMAP-Flaps: A Step by Step Guide of the Split Breast Technique

**DOI:** 10.3390/jcm9093030

**Published:** 2020-09-20

**Authors:** Kathrin Bachleitner, Laurenz Weitgasser, Amro Amr, Thomas Schoeller

**Affiliations:** Department of Hand, Breast, and Reconstructive Microsurgery, Marienhospital Stuttgart, 70199 Stuttgart, Germany; Laurenz.Weitgasser@vinzenz.de (L.W.); Amro.Amr@vinzenz.de (A.A.); Thomas.Schoeller@vinzenz.de (T.S.)

**Keywords:** perforator flap, IMAP-flap, split-breast, autologous breast reconstruction, microsurgery, free flap, breast reconstruction, breast reduction, reduction-mammoplasty

## Abstract

Various techniques for breast reconstruction ranging from reconstruction with implants to free tissue transfer, with the disadvantage of either carrying a foreign body or dealing with donor site morbidity, have been described. In patients who had a unilateral mastectomy and offer a contralateral mamma hypertrophy a breast reconstruction can be performed with the excess tissue from the hypertrophic side using the split breast technique. Here a local internal mammary artery perforator (IMAP) flap of the hypertrophic breast can be used for reconstruction avoiding the downsides of implants or a microsurgical reconstruction and simultaneously reducing the enlarged donor breast in order to achieve symmetry. Methods: Between April 2010 and February 2019 the split breast technique was performed in five patients after mastectomy due to breast cancer. Operating time, length of stay, complications and the need for secondary operations were analyzed and the surgical technique including flap supercharging were described in detail. Results: All five IMAP-flaps survived and an aesthetically pleasant result could be achieved using the split breast technique. An average of two secondary corrections to achieve better symmetry were necessary after each breast reconstruction. Complications included venous flap congestion, partial flap necrosis and asymmetry. No breast cancer recurrence was recorded. An overall approval of the surgical technique among patients was observed. Conclusions: The use of the contralateral breast for unilateral total breast reconstruction represents an additional highly useful technique for selected patients, is safe and reliable results can be achieved. Although this technique is carried out as a single-stage procedure, including breast reduction and reconstruction at the same time, secondary operations may be necessary to achieve superior symmetry and a satisfying aesthetic result. Survival of the IMAP-flaps can be improved by venous supercharging of the flaps onto the thoracoepigastric vein.

## 1. Introduction

Flaps used for breast reconstruction generally need to be soft and pliable in order to resemble breast tissue and to achieve aesthetically pleasing results. Furthermore, the donor tissue needs to be well-vascularized in order to be a reliable flap and should have an acceptable donor-site morbidity.

One of the first attempts to increase breast volume was made by Czerny [1], when he augmented a volume insufficient breast with a transferred lumbar lipoma in 1895. Since then, further milestones in breast reconstruction with autologous tissue have been achieved. Breast reconstruction using microsurgical techniques now represents a gold standard and is commonly performed today. Perforator flaps such as the free deep inferior epigastric perforator (DIEP) flap, first described by Isao Koshima in 1989 [2] are used now frequently for breast reconstruction around the globe.

Although microsurgical breast reconstruction is widely accepted today, older techniques and local flaps can still represent useful alternatives in selected cases. In patients where microsurgical techniques are contraindicated due to hypercoagulpathies or the inability to tolerate longer operations, or when no suitable autologous donor sites for free flaps can be found alternative local options for breast reconstruction can be useful. The use of contralateral breast tissue for unilateral autologous breast reconstruction in a two-stage procedure, was first described by Marshall et al. [3] in 1981. Here, a large pedicled transposition flap, which would normally be discarded during a breast reduction, is transferred to the volume deficient recipient site across the midline. Six weeks later, the broad pedicle is divided and final adjustments are made to both breasts in order to achieve symmetry [4]. This technique was modified by Schoeller et al. [5] who described a single-stage procedure in 2001. Another case report on splitting the breast along its longitudinal axis was published by Darius et al. [6] in 2008. Recently, Torres et al. [7] reported the successful implementation of the split breast technique in a series of seven patients. Overall, only a hand full of studies including a small group of patients has been published so far. To our knowledge, the underlying anatomy of the split breast technique in order to reliably perform unilateral breast reconstructions with this technique as well as options for venous supercharging has not been discussed and published so far.

The local flap used in the split breast technique is an internal mammary artery perforator (IMAP) flap, which is supplied by the anterior perforating branches of the internal mammary artery. The perforators of the internal mammarian artery pass through the intercostal spaces, penetrate the pectoralis major muscle and pass through the fascia before arborizing in a lateral direction in the subcutaneous tissue of the breast. The IMAP-flap based on the sizable perforator of the fourth intercostal spaces reliably supplies a large enough angiosome of the skin inferior to the areola and superior to the inframammary fold which can be raised as a local flap for breast reconstruction. Average flap sizes of 14 × 4 cm are reported in the literature, which can be reliably perfused by a perforator with an average caliber of 1.3 mm [8]. The flap is drained through accompanying venae comitantes of the fourth intercostal perforator [9,10]. Venous congestion and consequent flap tip necrosis are a common risk of this technique and has earlier been described in the literature [4,5]. Since described in the literature, venous congestion and consequent flap tip necrosis are relatively common. Therefore, the authors elaborated venous supercharging of the flap as an option to avoid this complication. This can be anticipated to reduce the risk of venous congestion and flap tip necrosis, which has not been described to our knowledge so far.

This study demonstrates five clinical cases of unilateral autologous breast reconstruction with contralateral IMAP-flaps using the split breast technique. The aim of this study was to present a step-by-step operative guide and describe how flap perfusion and survival can be improved by venous supercharging.

## 2. Methods

Between April 2010 and February 2019, five patients underwent a unilateral autologous breast reconstruction with a contralateral local IMAP-flap using the split breast technique. All five patients had a preoperative work up, involving breast ultrasound and mammography to exclude breast cancer of the IMAP-flap recipient site. The operation was only carried out once contralateral breast cancer was definitively excluded and no genetic predisposition or strong family history for breast cancer were present. The decision to perform a unilateral breast reconstruction with a contralateral local IMAP-flap using the split breast technique was preoperatively discussed in our multidisciplinary breast cancer team meeting. Any genetic predispositions for breast cancer, positive strong family history, as well as a high likelihood of secondary carcinoma or recurrence were considered an exclusion criteria. Age, Body-Mass-Index BMI, type of carcinoma, the TNM-classification of malignant tumors, date of mastectomy, time between tumor removal and reconstruction, adjuvant therapy, secondary diagnosis, number of operations and operation time were recorded and retrospectively analyzed and evaluated.

### Operative Technique

**Figure 1:**
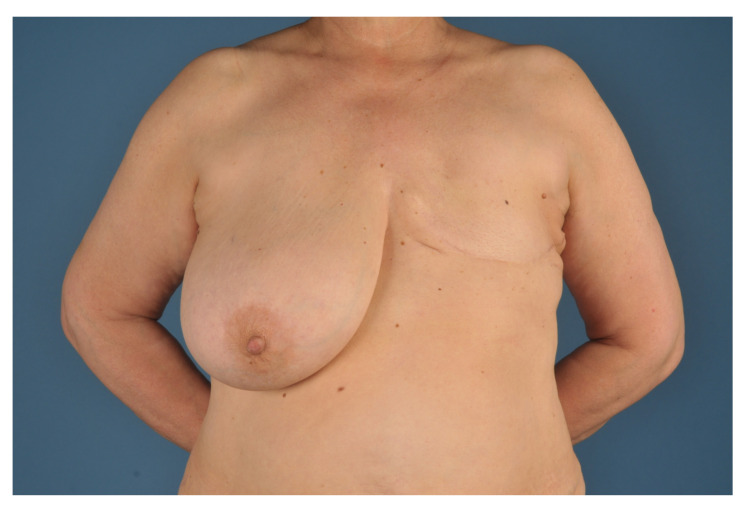
Selection criteria for the split breast technique: unilateral mastectomy defect and a ptotic and hypertrophic healthy donor breast on the contralateral side.

Preoperative patient selection is necessary. Ideal candidates for a unilateral autologous breast reconstruction with contralateral local IMAP-flaps using the split breast technique are females who had a unilateral mastectomy and have a very ptotic (Regnault Grade 3) and highly hypertrophic contralateral breast. Another mandatory requirement is the desire of the patient to reduce the size of the existing breast in order to achieve symmetry. As discussed, preoperative screening of the breast must be carried out to assure a tumor free status and avoid secondary carcinoma distribution through the reconstructive surgery itself (Figure 1).

**Figure 2:**
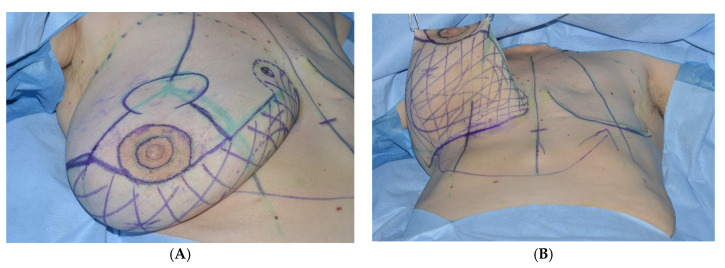
Preoperative Markings. (**A**) Incision planning according to a superiorly based reduction mammoplasty, (**B**) the dashed field represents the respective internal mammary artery perforator IMAP-flap angiosome.

2All patients are preoperatively marked in a standing position. The markings are similar to a superior based reduction mammaplasty. The inframammary fold, the axis of the breast and the position of the new areola, as well as the vertical limb are determined. Using a handheld Doppler ultrasound, the perforator, most frequently found in the fourth intercostal space, is verified and marked. The internal mammary artery perforator (IMAP)-flap is planned along its angiosome between areola and inframammary fold (Figure 2).

**Figure 3:**
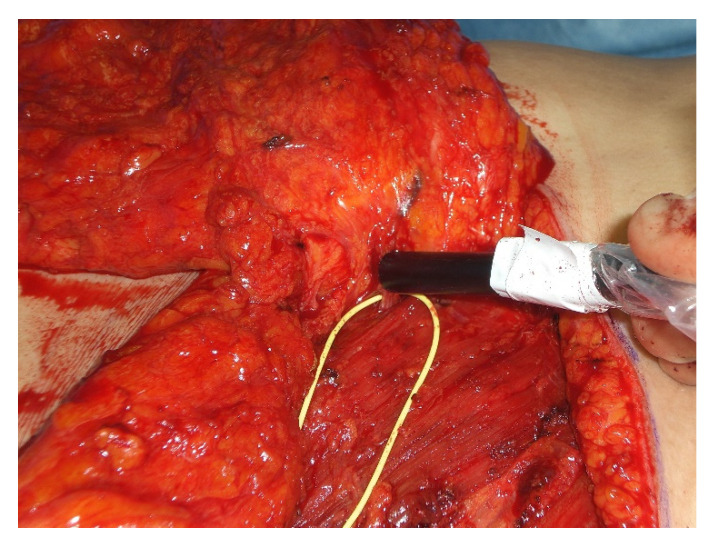
Preparation of the IMAP and evaluating its flow with a handheld Doppler.

With the patient in supine position under general anesthesia with both arms either tucked in or abducted, the operation begins with the superior based reduction of the contralateral breast and raising of the IMAP-flap. First, an incision in the inframammary fold extending laterally to the anterior axillary line onto the level of the pectoralis major fascia is made. Now the IMAP-flap is raised in a suprafascial plane by mobilization of the breast tissue in a cranial direction up to the level of the nipple-areola complex avoiding unnecessary medial preparation to prevent injury of the perforator. Once the IMAP is identified, a microsurgical perforator dissection through the pectoralis major muscle and into the intercostal space is performed to increase perforator mobility and avoid unnecessary twisting and tethering after rotation to the contralateral side (Figure 3).

**Figure 4:**
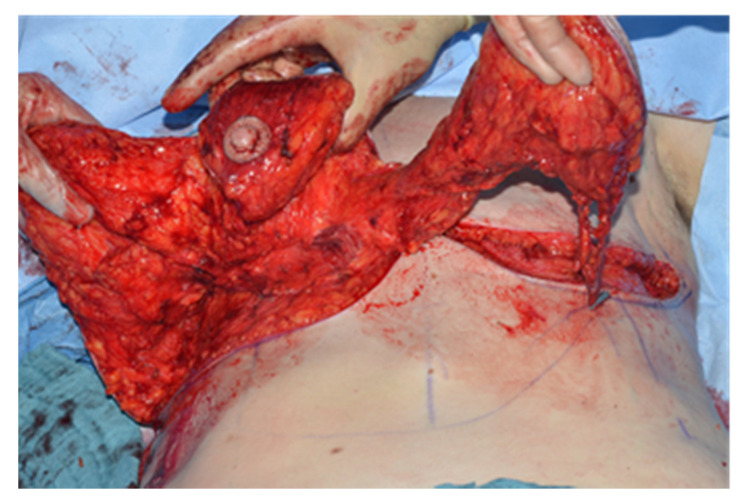
Dissected donor breast with its three pillars, Craniolateral pedicle, Craniomedial pedicle with the nipple-areola-complex, inferior pedicle perfused by the IMAP.

The breast is then “split” according to the pre-operative markings and reduced in a superior nipple areola complex based fashion. The blood supply of the inferior part of the breast comes from the fourth intercostal IMAP, while the nipple areola complex is still nourished by the first to third perforators through a superior medial or lateral pedicle. The superiomedial and lateral pillar are then used to close the donor site in similar fashion to a typical mamma reduction-plasty (Figure 4).

**Figure 5:**
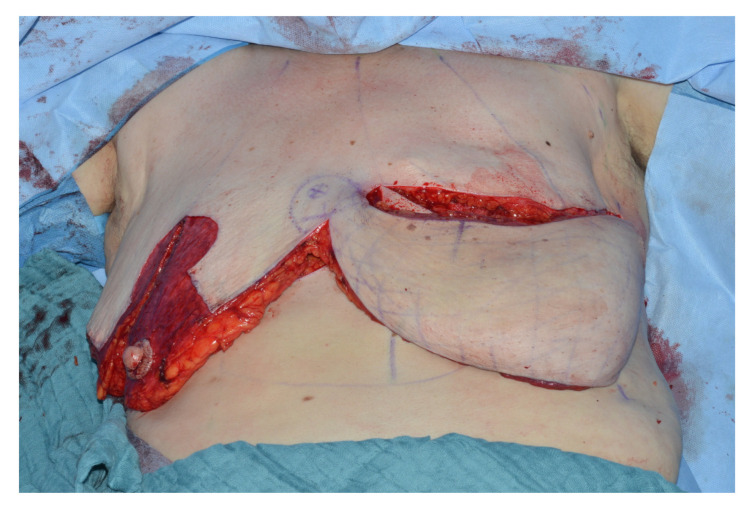
Rotation of the inferior part anticlockwise in a propeller fashion to the contralateral side.

The portion of the breast in between areola and inframammary fold representing the IMAP-flap which is normally discarded in a reduction mammoplasty, is then rotated 180° in a propeller like fashion to the contralateral recipient side, where the mastectomy scar is incised and a pocket for the split breast is created (Figure 5).

**Figure 6:**
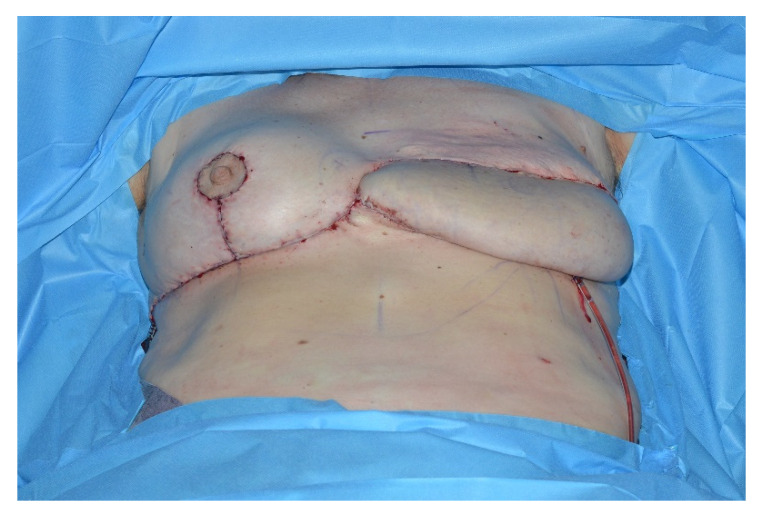
Immediate postoperative result.

This lipoglandulocutaneous flap is then adjusted in size and shape according to the mastectomy defect. The immediate postoperative result is shown in Figure 6.

**Figure 7:**
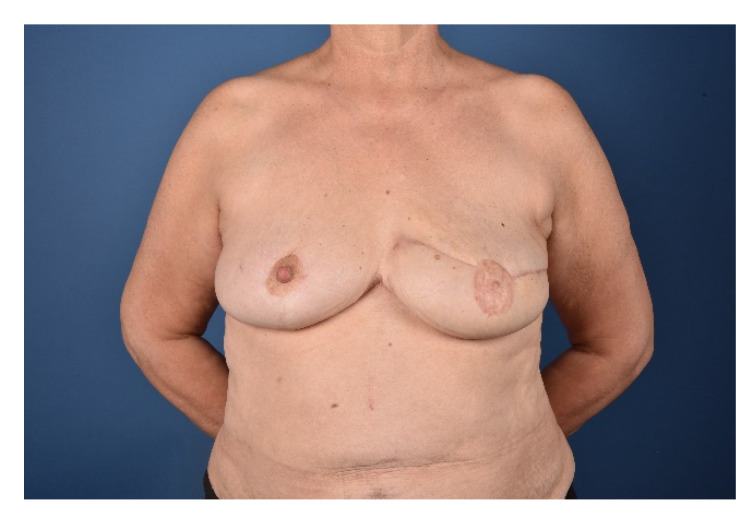
One-year postoperative result.

Secondary corrections to improve symmetry and adjust contour irregularities including reduction of medial bulging, liposuction and lipofilling, nipple reconstruction or tattooing of the areola are performed to achieve a pleasant aesthetic result. Special care needs to be taken to redefine the cleavage area and treat the present symmastia. The cleavage fold is recreated by aggressive tissue resection and quilting sutures to reduce the skin-sternum distance. Secondary corrections should not be performed earlier than three months after the initial flap operation to avoid compromising the flap perfusion. The final outcome one year after reconstruction is shown in Figure 7.

**Figure 8:**
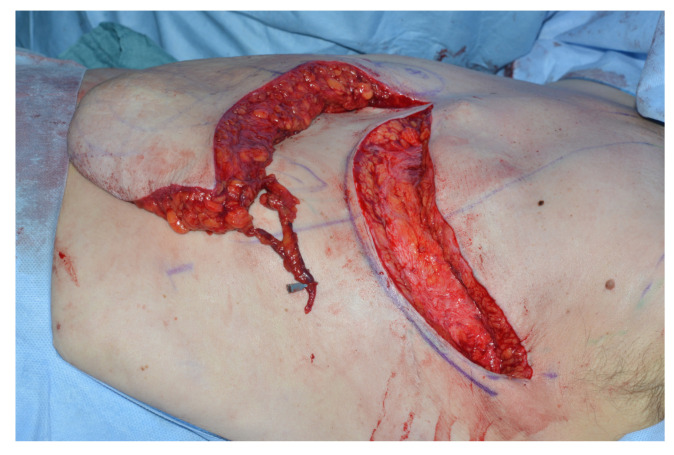
Dissection of a branch of the thoracoepigastric vein for venous supercharging of the distal third of the IMAP-flap.

To avoid venous congestion and necrosis of the flap tip supercharging of the IMAP-flap can be performed. In all cases, the thoracoepigastric vein is raised together with the IMAP-flap. Depending on flap tip congestion after flap inset, the thoracoepigastric vein incorporated in the IMAP flap tip can be reliably anastomosed to the thoracoepigastric vein on the contralateral side using microsurgical techniques (Figure 8).

## 3. Results

All five IMAP-flaps survived. One flap had a partial necrosis of the flap tip due to postoperative venous congestion. Other observed minor complications included fat necrosis, symmastia and medial bulging of the recipient side, asymmetry, and insufficient breast volume on the recipient side. In all five patients, a pleasant aesthetic result could be achieved. Minor secondary corrections including, flap trimming and thinning, liposuction, lipofilling, nipple reconstruction and tattooing were necessary. No donor site complications were observed. None of the patients had a cancer recurrence or developed a secondary carcinoma with an average follow up of 5 years. Details are shown in Table 1.

### Clinical Case Number 1

**Figure 9:**
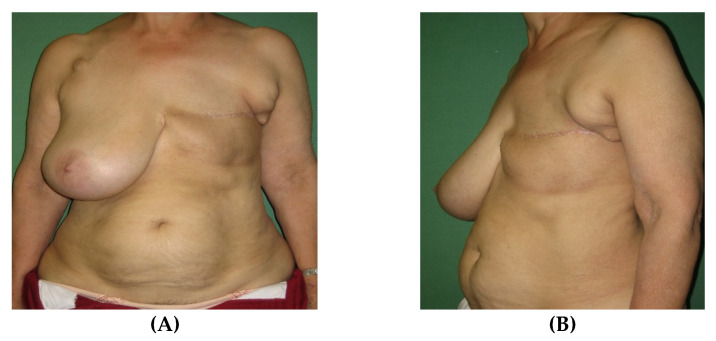

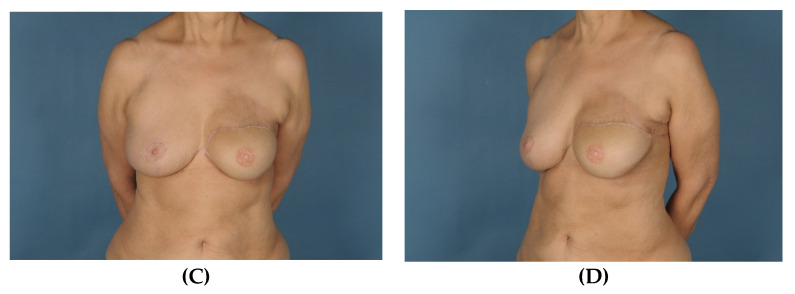
Pre- and one year postoperative results of Case 1. (**A**) On preoperative examination, front view. (**B**) On preoperative examination, side view. (**C**) On postoperative examination, front view. (**D**) On postoperative examination, side view.

Two years after unilateral mastectomy for an invasive ductal mamma carcinoma on the left side, a 64 years old patient presented with a hypertrophic and ptotic contralateral remaining breast. A unilateral reconstruction of the left side with a simultaneous reduction of the hypertrophic right side was planned using the split breast technique with an IMAP-flap. The flap was raised as described above and propelled 180° to the contralateral side to reconstruct the breast. Venous supercharging of the flap was performed by anastomosing the thoracoepigastric vein raised with the flap to the thoracoepigastric vein on the contralateral recipient side since the flap tip showed signs of venous congestion. Partial flap necrosis could be avoided and the flap survived entirely without any fat necrosis. Two final touch ups were necessary including correction of medial flap bulging by reintegrating and trimming the flap pedicle and reconstruction of the nipple areola complex (Figure 9).

## 4. Discussion

The success and reliability of microsurgical breast reconstructions together with their low rates of complications have more and more led to a neglect of “older” surgical techniques for breast reconstruction such as local flaps. The DIEP-flap represents a workhorse flap and is widely accepted as the gold standard for post mastectomy breast reconstruction. The reason for the DIEP-flap’s popularity is it’s low and well accepted donor site morbidity resembling an abdominoplasty, and the fact that abdominal tissue is often present in excess and is an ideal source of soft tissue for breast reconstruction as it offers similar skin quality, color, turgor as well as optimal pliability which enables easy molding and shaping.

In selected patients, alternatives such as local flaps for autologous breast reconstruction can come in handy. Sensitive patients may be reluctant regarding additional donor sites and their morbidity and ask for simple and easy solutions of autologous breast reconstruction with simultaneous symmetrization of the contralateral side in a single procedure. Especially older women with a unilateral mastectomy and a ptotic hypertrophic residual contralateral breast are ideal candidates for autologous unilateral breast reconstructions with the split breast technique.

The IMAP propeller flap used in the split breast technique has been widely accepted for various indications, such as deep sternal wound infections and chest wall reconstruction [11] or anterior neck reconstructions [12].

However, venous congestion and partial flap failures are common according to the literature [4,5,7] and the authors experience., especially in larger sized flaps. In order to avoid this complication, we started raising the thoracoepigastric vein together with the IMAP flap and anastomose it to the contralateral thoracoepigastric vein if flap tip congestion on table after flap inset is observed. To our knowledge this technique has not been described in detail in the literature yet. 

The anatomical basis for this venous supercharge is the reliable presence of the thoracoepigastric vein which ascends superficially on the anterolateral chest and abdominal wall bilaterally. It represents an important communication between the femoral and axillary vein draining into superior vena cava via the axillary vein and the inferior vena cava via the femoral vein. It, therefore, serves an anastomotic caval–caval link between the two, which becomes relevant in cases of portal hypertension where the thoracoepigastric vein can provide a collateral circuit of venous flow. The well-known consequence of this anastomotic circuit is the “caput medusae phenomenon”, caused by portal dilation of the paraumbilical and thoraoepigastric veins [13]. Postoperative inflammation of the thoracoepigastric veins is known as Mondor’s disease and can be sometimes encountered after breast surgery [14].

In the present case series, four out of five IMAP-flaps were venously supercharged by anastomosing the thoracoepigastric vein, which was raised together with the flap, with the thoracoepigastric vein on the contralateral side. Only the first flap was not supercharged since it did not seem to suffer from venous congestion after flap inset. This first case demonstrated a flap tip necrosis, all other IMAP-flaps, which were venously supercharged healed without venous congestion and necrosis of the flap tip.

In all described cases, minor additional secondary corrections were necessary to achieve a symmetric and pleasing aesthetic result. These were performed about three months after the initial operation to avoid compromising the IMAP flap perfusion. Here, the IMAP flap is adjusted to the contralateral donor breast. The symmastia is addressed with aggressive thinning and quilting sutures and the IMAP flap is reshaped accordingly with liposuction or lipofilling.

Sir Harold Gillies propagated one of the most simple and fundamental concepts in plastic surgery which is to “reconstruct like with like”. This concept represents the basis of the split breast technique where the ablated breast is reconstructed with excess lipoglandulocutaneous tissue from the contralateral side.

One of the risks that need to be considered when using the split breast technique is that potential cancerogenous breast tissue from the flap donor site is used for reconstruction. Therefore, thorough preoperative screening and exclusion of a genetic predisposition for breast cancer and a strong family history have to be excluded. The indication to perform a breast reconstruction with an IMAP flap using the split breast technique therefore should always be made in a multidisciplinary breast surgery team. According to Marshall et al., the risk of developing breast cancer on the contralateral breast does however not increase when splitting the breast [4]. Studies suggest a minimal risk of development of contralateral breast cancer within 0.30¨C.8% per year [15,16]. A slightly higher risk of contralateral breast cancer is reported for women with lobular—compared to those with ductal breast cancer [15,17]. In the present small cohort, we therefore, only included females with ductal breast cancer. Despite histological characteristics regular breast cancer, follow ups remain necessary in all patients after a breast reconstruction with the split breast technique. A cumulative risk of contralateral breast cancer of 63% when younger than 40 years of age at first diagnosis of breast cancer, compared to 20% for those who were older than 50 years of age was reported in females carrying a BRCA1 mutation [18]. Therefore, all patients with any genetic predisposition to breast cancer or aggressive tumors with a high risk of a cancer recurrence or breast cancer of the contralateral side must not receive reconstructions with this technique. Radiotherapy of a secondary breast cancer in a breast reconstructed with tissue from the contralateral side may also be limited since maximum radiation doses have already been applied in the primary cancer treatment.

The supercharged IMAP flap offers a unique additional reconstructive option and represents an acceptable alternative for autologous breast reconstruction in selected patients who had a unilateral mastectomy and offer a hypertrophic and ptotic healthy donor breast on the contralateral side. A simultaneous breast reduction of the donor breast and breast reconstruction of the contralateral side can be achieved. Besides, following the concept of reconstructing “like with like” and “breast with breast” a reduction of the hypertrophic donor breast improves the quality of life by re-balancing the weight and symmetry, therefore killing two birds with one stone. The indication to reconstruct a unilateral breast with a contralateral IMAP-flap using the split breast technique is relatively rare and is represented in the small sample size of the present study and comparable studies in the literature. The split breast technique offers a unique way of reconstructing a breast and at the same time avoiding additional donor flap donor sites or complex microsurgical reconstructions. Using venous supercharging of the IMAP-flaps in the split breast technique can improve flap survival and safety, therefore enabling reliable results with the need for relatively few secondary corrections, comparable to regular microsurgical reconstructions [19].

## Figures and Tables

**Table 1:** Patient characteristics and results.

	Patient 1	Patient 2	Patient 3	Patient 4	Patient 5
Age	69	67	64	58	76
BMI	34.0	30.5	31.0	32.3	28.3
Breast Cancer	Invasive Ductal Breast Cancer	Invasive Ductal Breast Cancer	Invasive Ductal Breast Cancer	Invasive Ductal Breast Cancer	Invasive Ductal Breast Cancer
TNM	pT2 M0 G2 N0 L0 V0 R0	pT1a G2 pN0 L0 V0 R0 M0	pT3b G1 N0 ER M0	PT2 M0 G2 N0 L0 V0 R0	pT2 G2 L0 V0 R0 M0
Date of Mastectomy	2008	2013	2014	2014	2018
Reconstruction	2010	2015	2016	2015	2019
Adjuvant Therapy	Chemo-, Radiotherapy, Hormone Therapy	Chemo-, Radiotherapy	Radiotherapy, Hormone Therapy	Chemo, Radiotherapy, Hormone Therapy	Radiotherapy, Hormone Therapy
Comorbidities	Art. Hypertension Asthma, Depression	DM Type II, Hep A,	Coronary Heart Disease, Art. Hypertension	Depression, Hypothyroidism	Art. Hypertension, Ex-Smoker
Complications	Venous Congestion, Partial Flap Loss, Asymmetry, Tenacious Scar	Contour Irregularities	Infected Fat Necrosis, Medial Bulging, Contour Irregularities	Contour Irregularities, Asymmetry	Contour Irregularities, Asymmetry
Amount of Secondary Corrections in GA	4	1	2	2	2
Operation Time (min)	327	221	210	213	252
